# Epidemiology and aetiology of maternal parasitic infections in low- and middle-income countries

**Published:** 2011-12

**Authors:** Tom Roberts, Courtney A. Gravett, Prasad Palani Velu, Evropi Theodoratou, Thor A. Wagner, Jian Shayne F. Zhang, Harry Campbell, Craig E. Rubens, Michael G. Gravett, Igor Rudan

**Affiliations:** 1 Centre for Population Health Sciences and Global Health Academy, The University of Edinburgh, Scotland, UK; 2 Global Alliance to Prevent Prematurity and Stillbirth (GAPPS), Seattle Children’s Hospital, Seattle, Washington, USA; 3 Department of Pediatrics, University of Washington, Seattle, Washington, USA; 4 Department of Obstetrics and Gynecology, University of Washington, Seattle, Washington, USA; * Joint first and joint senior authorship; **Correspondence to: **Igor Rudan Centre for Population Health Sciences University of Edinburgh Teviot Place Edinburgh EH8 9AG Scotland, UK igor.rudan@ed.ac.uk

## Abstract

**Background:**

There have been very few systematic reviews looking at maternal infections in the developing world, even though cutting maternal mortality by three quarters is United Nation’s Millennium Development Goal number five. This systematic review has two aims. The first is to present the prevalence of parasitic infections in the developing world over the last 30 years and the second is to evaluate the quality and distribution of research in this field.

**Methods:**

A systematic review of Medline, EMBASE and Global Health databases was undertaken using pre-determined search criteria. Three levels of quality criteria for exclusion of inadequate studies identified 115 out of initial 8580 titles. The data were extracted for 5 domains: worldwide pathogen prevalence, year of study, study setting, sample size and diagnostic test for each pathogen.

**Results:**

The initial search retrieved 8580 results. From these titles, 43 studies on malaria, 12 studies on helminths, 49 studies on *Toxoplasma gondii*, 7 studies on Chagas disease, 5 studies on *Trichomonas*, 1 leishmaniasis study and 1 study on trichinellosis were extracted for analysis. High prevalence of malaria was found in Gabon (up to 57%) India (55%), Cameroon (50%), Yemen (55%), Nigeria (up to 64%) and Ghana (54%). High prevalence of hookworm infections was found in Nepal at 78.8% and high values of *Ascaris lumbricoides* were found in Nepal, (56.2%), Kenya (52.3%) and Gabon (45.5%). High levels of *Schistosoma mansoni* were found in Zimbabwe (50%) and Tanzania (63.5%). The prevalence of active *Toxoplasma gondii* infection was found to be highest in India (27.7%).

**Conclusion:**

This study highlights the large burden of maternal parasitic infections globally. It may serve as a useful starting point for health policy development and research prioritization in this area.

With 5 years to go until 2015 and the end of the period to achieve Millennium Development Goals (MDGs) targets, it is debatable how many of the MDGs will reach the aims defined in 2000. MDG 5 aims to: “Reduce by three quarters the maternal mortality ratio” (1). However, between 1990 and 2005, in Sub-Saharan Africa especially, little progress had been made and in some countries the figures have even increased (1). In 1990, the Democratic Republic of Congo had a maternal mortality ratio (MMR) of 870 per 100 000 and by 2005 it had increased to 1100 per 100 000 (1). In addition, in Tanzania, which is a more stable sub-Saharan African country, the MMR also increased from 770 per 100 000 in 1990 to 970 per 100 000 (1). At the recent review of the MDGs in 2010, the UN managed to secure US$ 40 billion (€ 30 billion) for women’s and children’s health alone (2).

Maternal deaths are due to many causes including haemorrhage, hypertensive disorders, abortion related complications, infections and sepsis (3). Maternal infections contribute to about 10%-20% of these deaths (3). It is therefore imperative that there is up to date research on these topics. So far, there have been no systematic reviews of the data.

This review aims to summarise the research that has been undertaken in the past 30 years. It will look at the global distribution of research and the year in which the research was done. It will also look at how this research was undertaken, where the research was done, how many subjects were included in papers and what microbiological diagnostic methods were used. Finally the paper will set out an up-to-date picture of the prevalence of parasitic infections in the developing world over the last 30 years.

## METHODS

### Literature search terms

Initial searches were conducted to identify suitable keywords and MeSH headings to use in the final search (**Table 1**). The search strategy was prepared with an input from a librarian. Searches were conducted in parallel by two reviewers (using OVID) in the following databases on 1 August 2010: Medline (1950 to August Week 4 2010); EMBASE (1980 to 2010 Week 30) and Global Health (1973 to August 2010).

**Table 1:** Search terms used to identify published articles on the prevalence and aetiology of maternal infections in the developing world

exp Infection/
AND
exp Pregnancy/ OR exp Pregnancy Complications, Infectious/
AND
exp Developing Countries OR africa/ or africa, northern/ or algeria/ or egypt/ or libya/ or morocco/ or tunisia/ or “africa south of the sahara”/ or africa, central/ or cameroon/ or central african republic/ or chad/ or congo/ or “democratic republic of the congo”/ or gabon/ or africa, eastern/ or burundi/ or djibouti/ or eritrea/ or ethiopia/ or kenya/ or rwanda/ or somalia/ or sudan/ or tanzania/ or uganda/ or africa, southern/ or angola/ or botswana/ or lesotho/ or malawi/ or mozambique/ or namibia/ or south africa/ or swaziland/ or zambia/ or zimbabwe/ or africa, western/ or benin/ or burkina faso/ or cape verde/ or cote d'ivoire/ or gambia/ or ghana/ or guinea/ or guinea-bissau/ or liberia/ or mali/ or mauritania/ or niger/ or nigeria/ or senegal/ or sierra leone/ or togo/ or caribbean region/ or west indies/ or “antigua and barbuda”/ or cuba/ or dominica/ or dominican republic/ or grenada/ or guadeloupe/ or haiti/ or jamaica/ or martinique/ or “saint kitts and nevis”/ or saint lucia/ or “saint vincent and the grenadines”/ or central america/ or belize/ or costa rica/ or el salvador/ or guatemala/ or honduras/ or nicaragua/ or panama/ or latin america/ or mexico/ or south america/ or argentina/ or bolivia/ or brazil/ or chile/ or colombia/ or ecuador/ or french guiana/ or guyana/ or paraguay/ or peru/ or suriname/ or uruguay/ or venezuela/ or asia/ or asia, central/ or kazakhstan/ or kyrgyzstan/ or tajikistan/ or turkmenistan/ or uzbekistan/ or asia, southeastern/ or borneo/ or brunei/ or cambodia/ or east timor/ or indonesia/ or laos/ or malaysia/ or mekong valley/ or myanmar/ or philippines/ or thailand/ or vietnam/ or asia, western/ or bangladesh/ or bhutan/ or india/ or sikkim/ or middle east/ or afghanistan/ or iran/ or iraq/ or jordan/ or lebanon/ or syria/ or turkey/ or yemen/ or nepal/ or pakistan/ or sri lanka/ or far east/ or china/ or tibet/ or “democratic people's republic of korea”/ or mongolia/ or taiwan/ or atlantic islands/ or azores/ or albania/ or lithuania/ or bosnia-herzegovina/ or bulgaria/ or byelarus/ or “macedonia (republic)”/ or moldova/ or montenegro/ or romania/ or russia/ or bashkiria/ or dagestan/ or moscow/ or siberia/ or serbia/ or ukraine/ or yugoslavia/ or armenia/ or azerbaijan/ or “georgia (republic)”/ or indian ocean islands/ or comoros/ or madagascar/ or mauritius/ or reunion/ or seychelles/ or fiji/ or papua new guinea/ or vanuatu/ or guam/ or palau/ or “independent state of samoa”/ or tonga/

### Study inclusion and exclusion criteria

Studies were screened by title and then by abstract for relevance. Studies were deemed relevant if they provided information on the aetiology or epidemiology of parasitic infections in pregnant women in developing countries. These studies were then grouped according to pathogen studied, with some studies providing information on multiple pathogens. Studies providing information on the epidemiology of bacterial or viral infections in pregnant women were identified but not analyzed, as they were addressed in a separate review. Relevant English language papers were analyzed in this work, along with Chinese electronic databases, with the intention of translating and analyzing non-English papers, too. The inclusion criteria were:

***Subjects****:* Pregnant women at any stage of pregnancy or labour, including the puerperium (up to 42 days after labour);

***Study location****:* Low- and middle-income countries (as defined by the World Bank in 2010);

***Study design and sampling methods****:* No restrictions applied;

***Data collection****:* Only studies that provided evidence of parasitic infection using microbiological or serological test results were included;

***Results****:* Papers were selected if they provided information on the burden of a particular pathogen (the prevalence of a particular infection in pregnant women in the community over time/incidence) and/or the aetiology of parasitical maternal infections (prevalence of a specific pathogen/infection).

### Quality criteria

Papers were required to describe their samples and methods in detail, and provide microbiological or serological evidence of the aetiology of infection.

### Data extraction

Information on pathogen studied, sample population (pregnant women studied during pregnancy or at labour) and size, study setting, duration and type, microbiological/serological test used and results were extracted from abstracts and full papers for analysis.

### Data analysis

Epidemiology and aetiology of maternal parasitic infections were summarized according to the pathogen studied. Only pathogens with 5 or more studies reporting on its epidemiology and/or aetiology were analyzed. Median prevalence of each infection was calculated and trends in the prevalence of maternal infections were noted.

### Selection of studies

**Figure 1:**
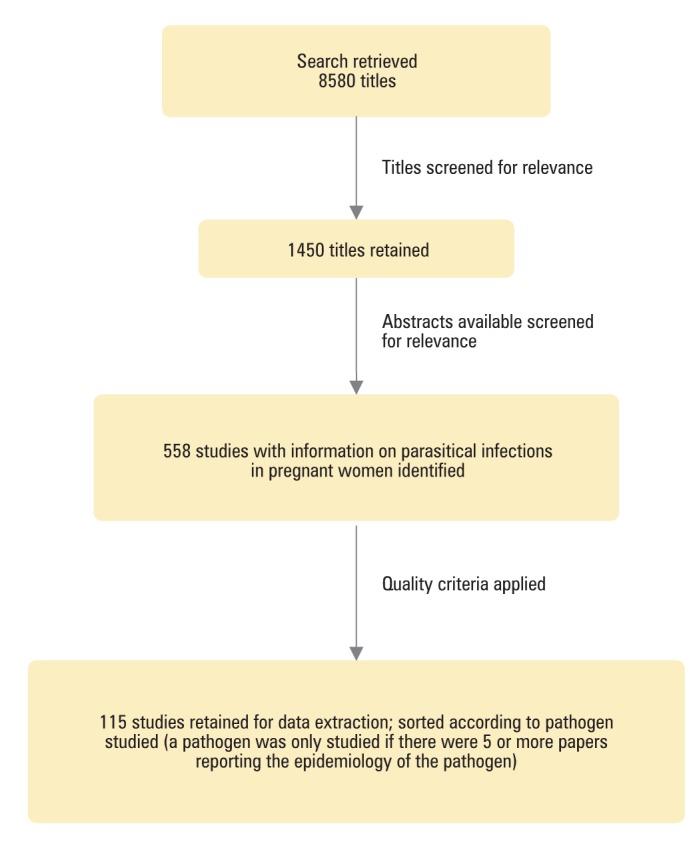
Summary of the literature search conducted.

The final search yielded 8580 relevant titles. **Figure 1** outlines the results of the search process and application of inclusion and exclusion criteria, resulting in the final panel of studies from which data was extracted.

Studies retained for data extraction (n=115) characterized the prevalence of 6 parasitic pathogens (malaria, helminths, *Toxoplasma gondii,* Chagas disease, trichinellosis and *Trichomonas vaginalis*) among pregnant women in developing countries, with 3 further reports providing secondary cross-sectional insights or reviews of the literature in this field, which were considered useful (4-118).

## RESULTS

### Prevalence of parasitical infections

**Figure 2:**
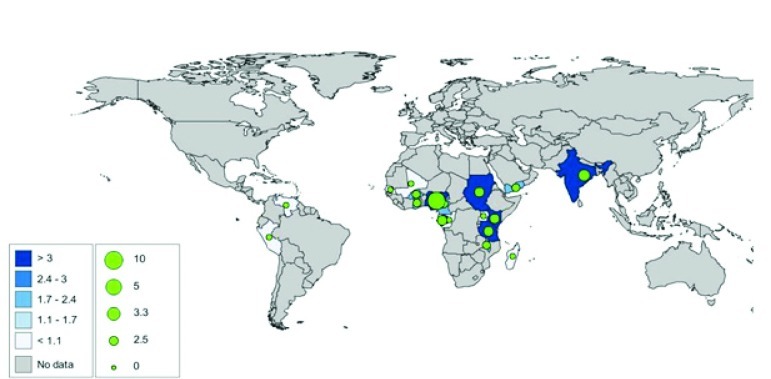
Geographical distribution of studies (n=43) reporting the prevalence of maternal malaria; “No data” in the legend refers to low- and middle-income countries only, as data from high-income countries were not the subject of this study.

**Figure 3:**
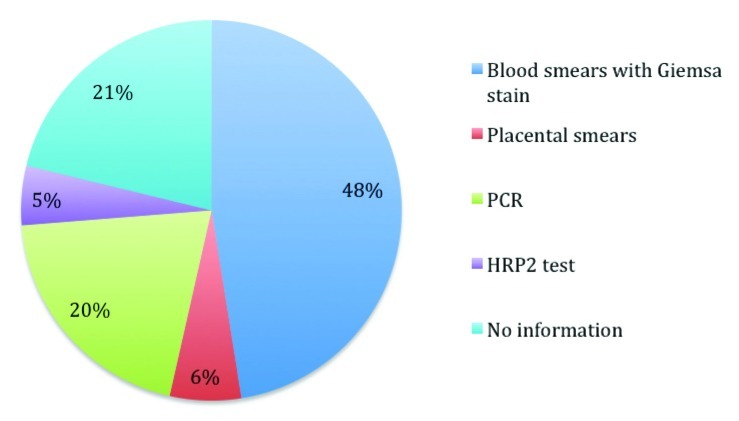
Techniques used to diagnose maternal malaria in the 43 studies identified. PCR – polymerase chain reaction, HRP2 test – histidine-rich protein 2.

**Figure 4:**
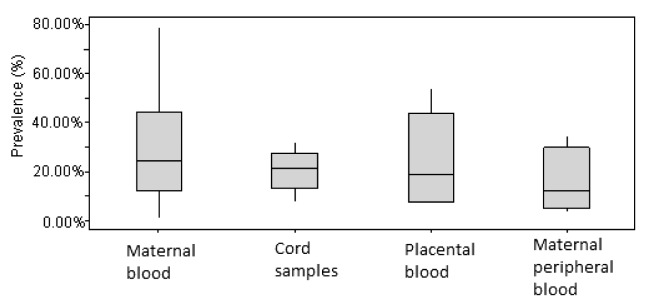
Box plot of malaria prevalence in different blood samples, reported by 43 relevant studies. The following number summaries are depicted in the boxplot: sample minimum, lower quartile, median, upper quartile, and sample maximum.

***Malaria.*** We identified 43 studies characterizing the prevalence of maternal malaria in 19 developing countries (**Supplementary Table 1**)(Supplementary Table 1). The features and results of these studies are summarised in **Table 2** and **Figures 2**,******3******and******4**.

**Table 2:** Distribution according to the size of population studied in 43 studies reporting maternal malaria prevalence

Size of population	Number (%)
0-500	28 (65%)
501-1000	6 (14%)
1001-1500	4 (9%)
1501-2000	0 (0%)
2001-2500	0 (0%)
2501-3000	1 (2%)
3001-3500	0 (0%)
3501-4000	1 (2%)
4001-4500	1 (2%)
4501-5000	0 (0%)
>5000	2 (5%)

The majority of studies had small sample sizes (**Table 2**), between 0-500 subjects and most of them were conducted in antenatal clinics or hospitals (82.7%), suggesting either awareness towards the need for antenatal screening for maternal malaria infection, or merely that it is much easier to recruit study subjects in health care facilities. The remaining studies (8.9%) were community-based and the study setting was not specified in 8.4%.

Blood smears with Giemsa stains were by far the most regularly used diagnostic test, accounting for 47% of all tests used to detect Malaria (**Figure 3**). Very high prevalenceof *Plasmodium vivax* (78.69%) was found in Brazil (28). Nine studies in seven different countries reported prevalence of over 50%: Gabon (57% and 54%) India (55%), Cameroon (50%), Yemen (55%), Nigeria (57% and 64%) and Ghana (54%) (26,29,39,46,47,49,51). The mean prevalence of malaria, regardless of the blood site taken from, was between 20%-30%, suggesting a degree of consistency between tests from different blood sites (**Figure 4**).

**Figure 5:**
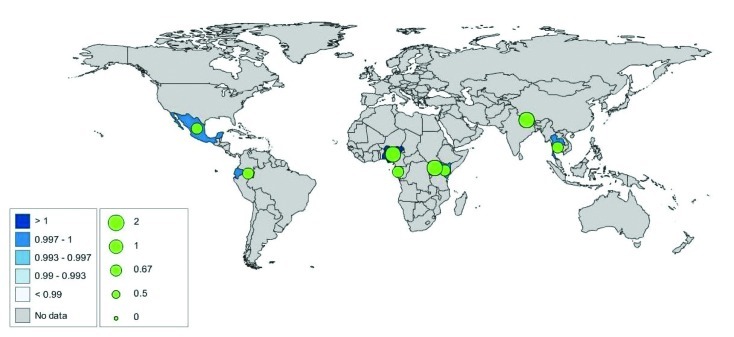
Geographical distribution of studies (n = 14) reporting the prevalence of maternal infection with helminths. “No data” in the legend refers to low- and middle-income countries only, as data from high-income countries were not the subject of this study.

**Figure 6:**
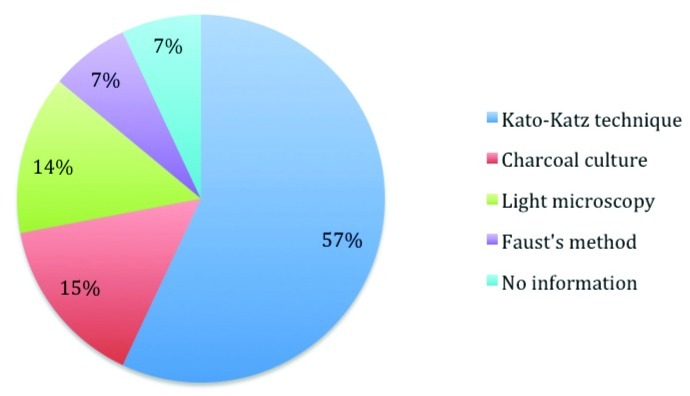
Techniques used to diagnose maternal infection with helminths in 14 studies identified.

**Figure 7:**
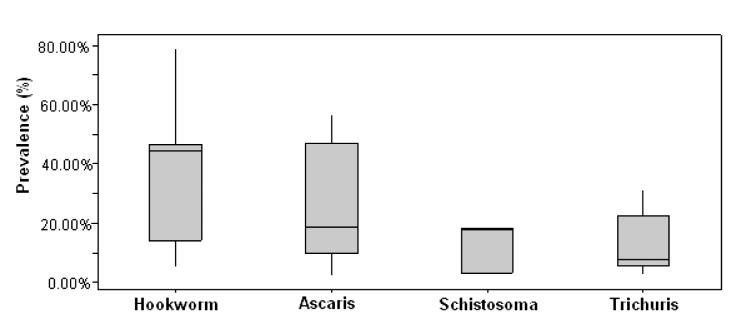
Box plot of the prevalence of maternal infections with helminths reported by the 14 relevant studies. The following number summaries are depicted in the boxplot: sample minimum, lower quartile, median, upper quartile, and sample maximum.

***Helminths.*** Fourteen studies characterizing the prevalence of maternal infections with helminths in 11 developing countries were identified (**Supplementary Table 2**)(Supplementary Table 2). The features and results of these studies are summarised in **Table 3** and **Figures 5**,******6** and **7**.

**Table 3:** Distribution according to size of population studied in 14 studies reporting maternal infection with helminth prevalence

Size of population	Number (%)
0-500	9 (69%)
501-1000	0 (0%)
1001-1500	1 (8%)
1501-2000	0 (0%)
2001-2500	1 (8%)
2501-3000	2 (15%)
3001-3500	0 (0%)
3501-4000	0 (0%)
4001-4500	0 (0%)
4501-5000	0 (0%)
>5000	0 (0%)

The majority of studies had small sample sizes (**Table 3**), between 0-500 subjects and most of them were conducted in antenatal clinics or hospitals (75%), suggesting either awareness towards the need for antenatal screening for maternal malaria infection, or merely that it is much easier to recruit study subjects in health care facilities. The remaining studies (18%) were community-based and the study setting was not specified in 9%.

Between the studies there was a generalised trend to use the Kato-Katz technique for examining stool samples (**Figure 6**), which is the gold standard for an epidemiological survey of helminth eggs (119). The prevalence of the different helminths varied across the studies. The most prevalent was hookworm, with a mean prevalence of 45%. The highest prevalence of hookworm infections was found in Nepal at 78.8% and high values were found in Uganda (44.5% and 45% in 2 studies), Tanzania (56.3%) and Kenya (39.5%) (12,16,18,19,24). For *Ascaris lumbricoides* relatively high prevalences were found in Nepal, (56.2%), Kenya (52.3%) and Gabon (45.5%) (11,18,19). *Trichuris trichiura* was least prevalent with a highest prevalence of 31% in Gabon and a mean value of 13% (11). High levels of *Schistosoma mansoni* were found in Zimbabwe (50%) and Tanzania (63.5%), but the prevalence in other countries was typically around 30% (10,24) (**Figure 7**).

**Figure 8:**
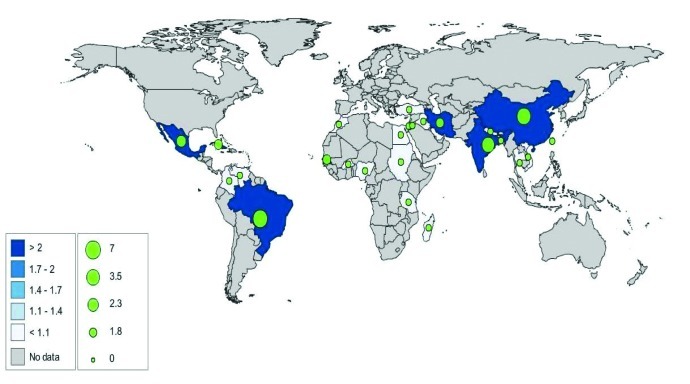
Geographical distribution of studies (n=49) reporting the prevalence of maternal infection with *Toxoplasma gondii*; “No data” in the legend refers to low- and middle-income countries only, as data from high-income countries were not the subject of this study.

**Figure 9:**
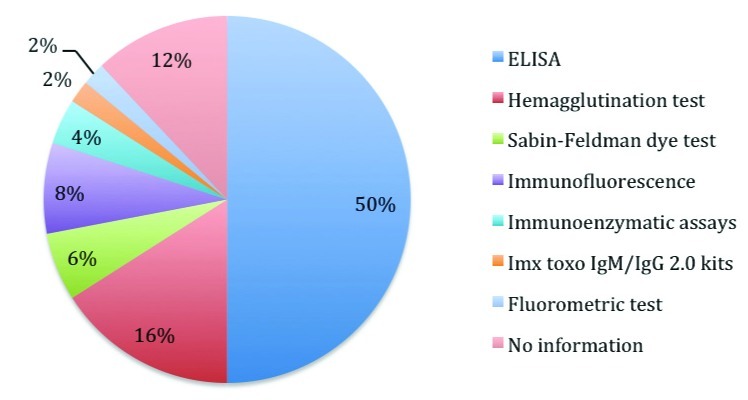
Techniques used to diagnose maternal infection with *Toxoplasma gondii* in 49 studies identified.

**Figure 10:**
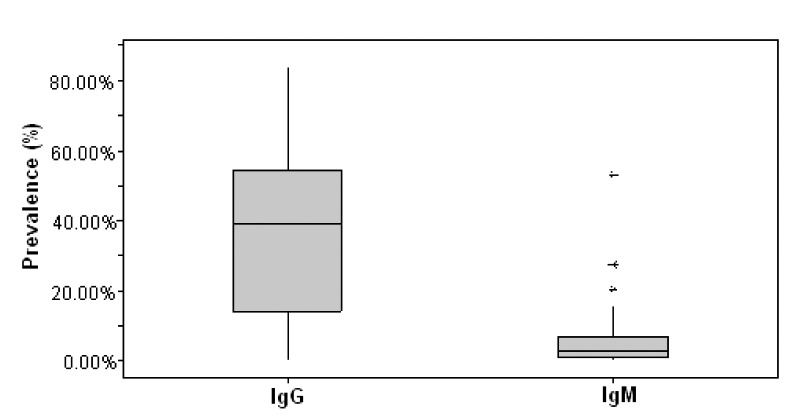
Box plot of maternal infections with Toxoplasma *gondii* prevalence reported by 48 relevant studies. The following number summaries are depicted in the boxplot: sample minimum, lower quartile, median, upper quartile, and sample maximum. Asterisks indicate outliers.

***Toxoplasma gondii.*** Forty-nine studies characterizing the prevalence of maternal infections with *T. gondii* in 26 developing countries were identified (**Supplementary Table 3**)(Supplementary Table 3). The features and results of these studies are summarised in **Table 4** and **Figures 8**,******9******and **10**.

**Table 4:** Distribution according to size of population studied in 48 studies reporting maternal infection with *Toxoplasma gondii* prevalence

Size of population	Number (%)
0-500	29 (60%)
501-1000	6 (13%)
1001-1500	4 (8%)
1501-2000	1 (2%)
2001-2500	2 (4%)
2501-3000	0 (0%)
3001-3500	0 (0%)
3501-4000	0 (0%)
4001-4500	1 (2%)
4501-5000	1 (2%)
>5000	4 (8%)

The majority of studies had small sample sizes (**Table 4**), between 0-500 subjects. Twenty five percent of the studies were conducted in antenatal clinics, hospitals, health care facilities or prenatal clinics. The remaining studies (2%) were community-based and the study setting was not specified in 75% of the studies.

The most commonly used test was ELISA, which is the gold standard for *T. gondii* analysis (**Figure 9**). The prevalence of both IgG and IgM was measured in 22 out of the 49 studies, useful for analysing current (IgM) and past (IgG) infections in pregnant mothers. The prevalence of active infection was low with a mean of 4% but there were high levels in India (27.7%), Mexico (20.7%) and Sudan (14.3%). The mean IgG (past infection) prevalence was 39%, highest prevalence in Brazil (75.1%) (**Figure 10**).

**Figure 11:**
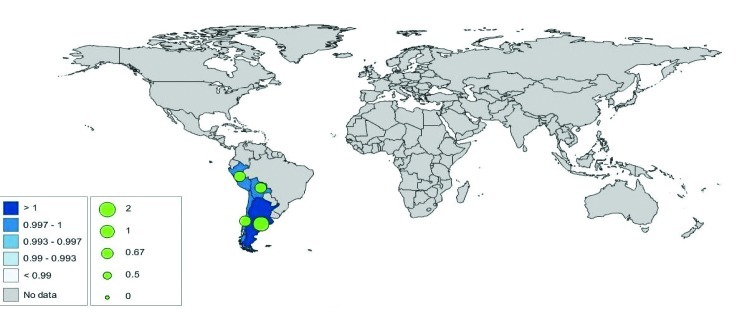
Geographical distribution of studies (n=7) reporting the prevalence of maternal infection with Chagas disease; “No data” in the legend refers to low- and middle-income countries only, as data from high-income countries were not the subject of this study.

**Figure 12:**
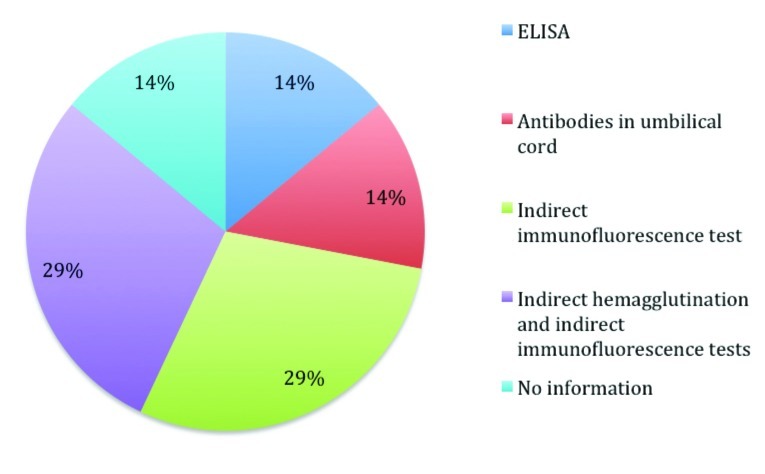
Techniques used to diagnose maternal infection with *Toxoplasma* g*ondii* in the 49 studies identified.

**Figure 13:**
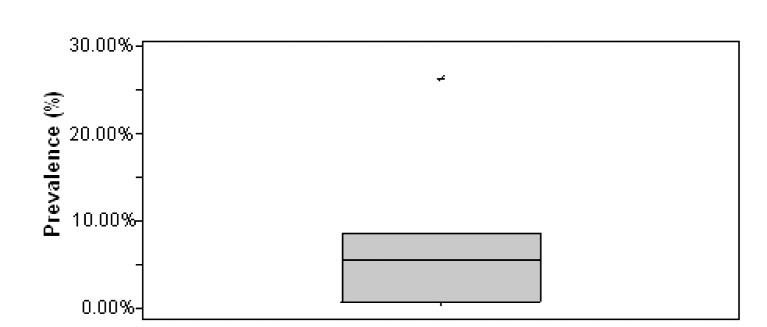
Box plot of maternal infections with Chagas disease prevalence reported by the 749 relevant studies. The following number summaries are depicted in the boxplot: sample minimum, lower quartile, median, upper quartile, and sample maximum. Asterisk outlines an outlier.

***Chagas disease.*** Seven characterizing the prevalence of maternal infections with Chagas disease in 7 developing countries were identified (**Supplementary Table 4**)(Supplementary Table 4). The features and results of these studies are summarised in **Table 5** and **Figures 11**,******12** and **13**.

**Table 5:** Distribution according to size of population studied in 7 studies reporting maternal infection with Chagas disease prevalence

Size of population	Number (%)
0-500	1 (14%)
501-1000	2 (29%)
1001-1500	0 (0%)
1501-2000	0 (0%)
2001-2500	3 (43%)
2501-3000	0 (0%)
3001-3500	1 (14%)
3501-4000	0 (0%)
4001-4500	0 (0%)
4501-5000	0 (0%)
>5000	0 (0%)

The majority of studies had small sample sizes (**Table 5**), between 0-500 subjects. Fourteen percent of the studies were conducted in hospitals, 29% in endemic and 29% in non-endemic areas. The remaining studies (14%) were conducted in parturient and the study setting was not specified in 14% of the studies.

The most commonly used tests were indirect immunoflouresence and indirect haemogluttination tests (**Figure 12**). Chagas disease had a low mean prevalence of around 7.2% in pregnant women but a study in Bolivia (9) reported a prevalence of 26.3% (**Figure 13**).

***Trichinellosis*.** There was only one study looking at trichinellosis (**Supplementary Table 5**)(Supplementary Table 5). Because of the scarcity of studies it is hard to make any inference to trends, so more research needs to be done in this area.

***Trichomonas vaginalis*.** We found only four studies focused on trichomoniasis (**Supplementary Table 6**)(Supplementary Table 6). Because of the scarcity of studies it is hard to make any inference to trends and more research is needed.

## DISCUSSION

### Prevalence of parasitic infections

Our search of published literature relevant to the aetiology and epidemiology of maternal parasitic infections in the developing world provided detailed epidemiological information on 6 maternal infections. These 6 parasitical maternal infections were most extensively studied, suggesting that these infections have a high burden on pregnancy outcomes in the developing world. These infections also have potential adverse effects on neonates.

Malaria in its own right is a major contributing factor towards maternal deaths worldwide. It causes severe anaemia and can affect newborn‘s birth-weight and long-term survival (119). For this reason, it is recommended that research conducted in South and Central America, especially Brazil where the prevalence of *P. vivax* was so high (78.69%) to be expanded (28). Further research should also be concentrated on regions classified as high risk but with a current lack of data like Democratic Republic of Congo, Angola and Zambia, from where no evidence of research was found. Finally, prevention strategies should be encouraged in the maternal populations from the countries with evidence of very high malaria prevalence, namely Gabon, India, Cameroon, Yemen, Nigeria and Ghana (26,29,39,46,47,49,51).

Hookworm infections were found to be well studied, which is very promising as these parasites cause anaemia during pregnancy and have been noted to increase the maternal and child mortality rates (120). However more studies looking at *Schistosoma* would definitely be recommended as there were only 4 studies in pregnant women, despite WHO’s statement that *Schistosoma* is “second only to malaria in public health importance” and pregnant women are one of the important at risk groups (121). Specific countries of note for further investigation and preventative measures would be Nepal for hookworm (78.8%) and *A. lumbricoides* (56.2%) (19); Tanzania for hookworms (56.3%) and *Schistosoma mansoni* (63.5%) (24); Kenya for hookworms (39.5%) and *A. lumbricoides* (52.3%); the Gabon for *A. lumbricoides* (45.5%) and *Trichuris trichuria* (31%); and finally for *S. mansoni* (50%) (10,11,18).

Chagas disease is important because of its potential to cause mortality in all age-groups. It can be transmitted vertically so that the knowledge of maternal prevalence is important (122). Although this review has shown studies in South America, the disease is no longer confined to just South America, as with Latino American emigration there has been a movement of the disease across the world. Therefore global research in areas of high Latino American immigration would be recommended to assess the extent this emigration has had (123). More funding in Bolivia would be recommended to assess the nature of the 26.3% prevalence of maternal infection and its effects on maternal and neonatal health (9).

There is a need for more studies on *Leishmania* would also be recommended from the results. Although not much evidence can be taken from the single study from Brazil (124), pregnant woman tended to have more severe *Leishmania* than the non-pregnant infected subjects and there was a possible link with increased problems with pregnancy (124).

### Strengths and limitations

To our knowledge, this is the first review that summarises the epidemiology of maternal parasitic infections in the developing world. The search strategy devised was sensitive and specific, which allowed for a comprehensive review of available literature on this topic. The information generated in this review can be used to guide public health policy and the allocation of resources within local governments and by the international community towards improving maternal health. Although the search did not exclude non-English papers, we did not search some of the databases where we would have expected a higher concentration of foreign language papers (e.g. LILACS). Although the databases used were very extensive, especially in the case of EMBASE and Medline there is a high chance that important papers could have been recovered from smaller, more specialist databases.

This study could be further improved by analysing non-English studies conducted in francophone parts of Africa (in French), South America (in Spanish) and in China (in Chinese), which could be accessed from appropriate databases. Reviewing non-English articles may assist in defining the epidemiology of pathogens for which we managed to identify few (<5) studies, as well as providing more robust data on the pathogens presented in this review. In addition, searching grey (unpublished) literature or contacting health officials and researchers in the field may also yield more country specific data on the subject, thus enabling more targeted and context-specific public health measures.

### Recommendations and future work

This systematic review highlights the quantity of maternal parasitic infection research and, to a lesser extent, quality of research that has been achieved over the last 30 years. This paper would therefore be useful to decision makers, especially in light of the US$ 40 billion (€ 30 billion) pledged at the last UN’s MDG summit, to help them assess where best to implement resources for research. It could also be a useful tool to measure how good the data are for each individual parasite and in assessing what areas of the world have been neglected in terms of research. With a full picture where different maternal infections occur, it could be used as a tool to target research and could ultimately lead to big leap forward maternal infections knowledge, therefore helping in the fight towards cutting down maternal infections.

### Conclusion

No mother should die during childbirth and every step should be taken to stop this happening. This review is a first step, in a long chain of events, trying to prevent maternal mortality. If research and knowledge is channelled into the right areas and decision makers have accurate knowledge regarding maternal infections then resources can be allocated to those areas that need it most.

Researchers have a responsibility to reflect on which part of the world current knowledge is stemming from and should look retrospectively to see how much research has been done in the past. By doing this, the public health community can positively expand research into areas of the world and into diseases where that information is lacking. And with this information, informed and important decision can be made about the factors that affect maternal mortality most and maybe, just maybe, Millennium Development Goal 5 can be achieved.
