# Supplementary material for: Epidemiology and aetiology of maternal parasitic infections in low- and middle-income countries
Source: J Glob Health. 2011 Dec;1(2):189–200. (PMC3484768)
Supplement: Supplementary Table 1 [file jogh-01-189-s001.pdf]

Supplementary Table 1. Summary of data extracted from studies reporting prevalence of maternal malaria infection (n=43)

Author	Country	Pathogen	Prevalence %	Numbers in study	Study setting	Tests used	Study dates
Gomez et al (25)	Venezuela	Malaria	27.4%	449	Hospital	0	2005-2006
Bouyou-Akote et al (26)	Gabon	Malaria: maternal peripheral	34.40%		Hospital		
		Malaria: placental	53.60%				
		Malaria: cord blood	18.20%				
Akanbi et al (27)	Nigeria	Malaria	41.80%	262	Antenatal clinic	Thick blood smears	2009
Chagas et al (28)	Brazil	Malaria: P. falciparum,	20.56%	417	Rural clinic	0	2009
		P. vivax	78.69%				
		Cases where both are present	0.75%				
Aribodor et al (29)	Nigeria	Malaria	64.40%	500	No information	Giemsa stain and microscopy	2005-2006
Adam et al (30)	Sudan	Malaria: acute infection	5.50%	236	Hospital	0	2009
		Malaria: chronic infection	2.10%				
Nwonwu et al (31)	Nigeria	Malaria	29.00%	198	Tertiary health institution	0	2009
Clerk et al (32)	Ghana	Malaria	47.00%	3642	Antenatal clinic	Giemsa stain and microscopy	June 2004 and July 2006.
Kabanywanyi et al (33)	Tanzania	Malaria	8.00%	413	Hospital	0	2008
Uneke et al (34)	Nigeria	Malaria	19.70%	300	No information	Histidine-rich proteins-2 RDT	2008
Parekh et al (35)	Peru	Malaria 2004	8.10%	1463	Tertiary health institution	Giemsa stain and microscopy	2004 and 2005.
		Malaria 2005	6.60%				
Coulibaly et al (36)	Burkina Faso	Malaria	32.20%	295	Antenatal clinic	Giemsa stain and microscopy	May-07
		Malaria	11.80%	288	Antenatal clinic	Giemsa stain and	Dec-07

						microscopy	
Tarimo et al (37)	Tanzania	Malaria	23.70%	395	Antenatal clinic	Blood smear	2007
Lukuka et al (38)	Congo	Malaria (Plasmodium falciparum)	21.00%	196	Antenatal clinic	Giemsa stain and microscopy	September and November 2004
Idowu et al (39)	Nigeria	Malaria	57.40%	466	Hospital		2006
Mockenhaupt et al (40)	Ghana	Malaria	19.00%	839	No information	Microscopy	2006
			34.00%			HRP2 test	
			54.00%			PCR	
Malhotra et al (41)	Kenya	Malaria (Falciparum malaria)	10.40%	632	Hospital	RTQ-PCR	2006
Bassiouny et al (42)	Yemen	Malaria	6.16%	276	Hospital	Giemsa stain and microscopy	March–August 2001
Adam et al (43)	Sudan	Malaria	11.90%	142	No information	Microscopy	August 2003 and July 2004
			32.00%	.		PCR	
Mwapasa et al (44)	Malawi	Malaria	24.30%	480 (HIV positive)	Hospital	Placental histopathology	2004
Elghazali et al (45)	Sudan	Malaria	13.70%	744	Antenatal clinic	Giemsa stain and microscopy	October 2003-May 2004
Bouyou-Akotet et al (46)	Gabon	Malaria	57.00%	311	Hospital urban	Thick blood smears	April 1995 to September 1996
Assabri et al (47)	Yemen	Malaria	55.00%	260	No information	Thick and thin blood films Giemsa stains and microscopy	2002
van Eijk et al (48)	Kenya	Placental malaria	19.00%	5093	Antenatal clinic	Thick and thin blood films Giemsa stains and microscopy	June 1996 and March 1999
	.	Maternal peripheral parasitaemia	15.20%				
Zhou et al (49)	Cameroon	Malaria	26% (1st 3 months of pregnancy)	719	Rural	Blood thick and thin films were prepared, stained with Diff-Quick reagents	1995–1998
			9% (1st 3 months of pregnancy)		City		

			50% (4th month of pregnancy)		Rural		
			20% (4th month of pregnancy)		City		
Cot,M et al (50)	Madagascar	Placental malaria	8.10%	2989	Hospital	Thick and thin blood films Giemsa stains and microscopy	June 1996 to May 1997
Singh et al (51)	India	Malaria	55% (88% Plasmodium falciparum, 12% P.Vivax)		Antenatal clinic	No information	1997-1998
Tobian et al (52)	Kenya	Malaria		102	No information		2000
		Plasmodium falciparum	3.4% (maternal peripheral blood)			PCR	
			0% (maternal peripheral blood)			Microscopy	
			48% maternal-blood samples			PCR	
			25% maternal-blood samples			PCR	
			24% maternal-blood samples			PCR	
		Plasmodium falciparum	32% cord-blood samples			PCR	
		Plasmodium malariae	23% cord-blood samples			PCR	
		Plasmodium ovale	21% cord-blood samples			PCR	
Kasumba et al (53)	Uganda	Malaria	8.6% peripheral blood	No information	Hospital	Smear	2000
			6.7% placental blood				
Singh et al	India	Malaria	17.6%	456	Rural	0	2005

(54)			(plasmodium falciparum 64% and Plasmodium vivax 36%)				
Egwunyenga et al (55)	Nigeria	Malaria	21.6% (plasmodium falciparum	1095	Hospital		
Steketee et al (56)	Malawi	Malaria	44.50%	4127	Antenatal clinic	Blood smear	1996
Okonofua et al (57)	Nigeria	Malaria	12.00%	91	Antenatal clinic	Blood smear	1996
Singh et al (58)	India	malaria	0.00%	145	No information		1995
Gazin et al (59)	Burkina Faso	Malaria (high season)	6.50%	1040	Hospital		1994
		Malaria (low season)	24.50%				
Singh et al (58)	India	Malaria	55.00%	274	No information		1997-1998 (malaria epidemic)
Ibhanesebhor et al (60)	Nigeria	Malaria	45.19%	312	No information	Placental smear	August 1989 and November 1989
Bako et al (61)	Nigeria	Malaria	33.90%	437	Hospital	Placental histology, Maternal packed cell volume and thick blood films	24 July 2007 and 12 January 2008
Agomo et al (62)	Nigeria	Malaria	7.70%	1084	No information	Blood smears stained with Giemsa	2009
Kayentao et al (63)	Mali	Malaria	17.10%	261	No information	0	2007
			42.30%	192	No information		
N'Dao et al (64)	Senegal	Malaria	9.50%	8270	Urban hospital	0	August 1998 to December 1999
Akum et al (65)	Cameroon	Malaria	7.8% cord	770	No information	Light microscopy using blood samples	June 1999 and September 2001
			32.8% maternal blood				

			33.7% placental biopsies				
Bouyou-Akotet et al (46)	Gabon	Malaria	57.00%	311	No information	Thick blood smears	2003
