# Supplementary material for: Epidemiology and aetiology of maternal parasitic infections in low- and middle-income countries
Source: J Glob Health. 2011 Dec;1(2):189–200. (PMC3484768)
Supplement: Supplementary Table 2 [file jogh-01-189-s002.pdf]

Supplementary Table 2. Summary of data extracted from studies reporting prevalence of maternal infection with helminths (n=14)

Author	Country	Pathogen	Prevalence	Number in study	Study setting	Test used	Date of study
Patana et al (10)	Zimbabwe	Schistosoma haematobium and Schistosoma mansoni	50.00%	299	Antenatal clinic	Urine and stool specimens for Schistosoma and 5ml of blood for HBV	0
		HBV	2.00%				
Adegnika et al (11)	Gabon	Ascaris lumbricoides	45.5% pregnant vs 32.5% non-pregnant	210	Hospital	Stool samples examined using the Kato-Katz technique	October 2003 and January 2004
		Trichuris trichiura	31% pregnant vs 12.5% non-pregnant				
Muhangi et al (12)	Uganda	Hookworm	44.50%	2507	Antenatal clinic	Kato–Katz method and charcoal culture for Strongyloides	April 2003 and November 2005.
		Mansonella perstans	21.30%				
		Schistosoma mansoni	18.30%				
		Strongyloides	12.30%				
		Trichuris	9.10%				
		Ascaris	2.30%				
		Asymptomatic Plasmodium Falciparum Parasitaemia	10.90%				
		HIV	11.90%				
Shah et al (13)	Nepal	Hookworm	46.50%	112	Antenatal clinic	Light microscopy of stool samples	May 2001 to July 2001
Ozumba et al (14)	Nigeria	Helminthic infection (the two below only two types identified	11.80%	161	Antenatal clinic	Kato thick smear technique	July and December 2000
		Ascaris lumbricoides	8.70%				
		Trichuris trichuria	3.10%				
Rodriguez-Garcia et al (15)	Mexico	Giardia lamblia	65.80%	207	No information	Faust's method with 3 stool samples	0
		Ascaris lumbricoides	13.90%				
Ndibazza et al (16)	Uganda	hookworm	45.00%	2507	Hospital	Kato-Katz method for ova and with use of charcoal culture for	April 2003 through

						Strongyloides species	November 2005
		Schistosoma mansoni	18.00%				
Liabsuetrakul et al (17)	Thailand	Ascaris	10.30%	1063	Hospital		
		Hookworm	5.70%				
		Trichuris	6.30%				
van Eijk et al (18)	Kenya	Ascaris lumbricoides	52.30%	390	Community	Microscopically examined for geohelminth infection using a modification of the formol-ether and ethyl acetate by concentration technique and by Kato-Katz method	0
		Hookworm	39.50%				
		Trichuris trichiura	29.00%				
Navitsky et al (19)	Nepal	Hookworm	78.80%	292	Rural	Kato-Katz method and modified Harada-Mori method	June 1995 through July 1996
		Ascaris lumbricoides	56.20%				
		Trichuris trichiura	7.90%				
		Strongyloides stercoralis	2.00%				
Guerra et al (20)	Brazil	Ascaris lumbricoides	19.00%	481	Urban	0	April to October of 1988
		ancilostomideos	16.70%				
		Trichuris trichiura	15.90%				
Brooker et al (21)	Sub-Saharan Africa	Hookworm	37.7 million			GPW3.0b population estimates and country-specific age- sex structures	0
Egwunyenga et al (22)	Nigeria	Malaria parasites	816 (38.8%)	2014	Hospital	Kato-Katz Method	1st February 1997 and 31st January 1998
		Ascaris lumbricoides	19.10%				
		Hookworm	14.20%				
		Trichuris trichiura	7.00%				
		Schistosoma mansoni	3.40%				
		Enterobius vermicularis	2.00%				

		Hymenolepis sp.	1.60%				
		Taenia sp.	1.00%				
Weigel et al (23)	Ecuador	E.Histolytica	80.00%	91	Hosptial	Kato-Katz Method	0
		E.Coli	62.00%				
		Giardia	22.00%				
		Ascaris	21.00%				
		Trichuris	5.00%				
