# Supplementary material for: Epidemiology and aetiology of maternal parasitic infections in low- and middle-income countries
Source: J Glob Health. 2011 Dec;1(2):189–200. (PMC3484768)
Supplement: Supplementary Table 3 [file jogh-01-189-s003.pdf]

Supplementary Table 3. Summary of data extracted from studies reporting prevalence of maternal infection with *Toxoplasma gondii* (n=49)

Author	Pathogen	Country	Prevalence %	Numbers in study	Study setting	Tests used	Duration of study
Wanachiwanawin et al (66)	Toxoplasma	Thailand		1669 838 HIV positive 831 HIV negative	Antenatal clinic	Solid-phase enzyme-linked immunosorbent assay for IgG and capture antibody enzyme immunoassay for IgM	2001
	IgG		53.7% HIV seropositive				
	IgG		5.3% non-HIV infected				
	IgM		0.67% of IgG positive in HIV positive				
	IgM		4.5% of IgG positive in HIV negative				
Lopes et al (67)	Toxoplasma	Brazil		492	No information	Chemiluminescence	2009
	IgG		49.20%				
	IgM		1.2% of IgG				
Varella et al (68)	Toxoplasma	Brazil	0.48%	41112	No information	0	1998-2005
Khurana et al (69)	Toxoplasma	India		300	No information	ELISA	2010
	IgG		15.30%				
	IgM		3.00%				
Vaz et al (70)	Toxoplasma	Brazil		20389	No information	Immunoenzymatic assays	01 April 2003-31 July 2004
	IgG		53.30%				
	IgM		3.26%				
Alvarado-Esquivel et al (71)	Toxoplasma	Mexico		439	Rural	Enzyme-linked immunoassays	2009
	IgG		8.20%				
	IgM		2.30%				

Maggi et al (72)	Toxoplasma	Albania		496	No information	0	October 2004 to March 2005
	IgG		48.60%				
	IgM		1.3% of IgG positive				
Barbosa et al (73)	Toxoplasma	Brazil	66.30%	190	No information	Microparticle enzyme immunoassay	March to December 2007.
	Toxoplasma	China		235			
Liu et al (74)	IgG		10.60%		Antenatal clinic	ELISA	2006
	IgM		0.00%				
Ribeiro et al (75)	Toxoplasma	Brazil		831	No information	0	2008
	IgG		75.10%				
	IgM		2.00%				
Rosso et al (76)	Toxoplasma	Colombia		955	Healthcare facility	Microparticle enzyme immunoassay	July 22, 2005 to December 31, 2005
	IgG		45.80%				
	IgM		2.80%				
Abdi et al (77)	Toxoplasma IgG	Iran	44.60%	553	No information	Indirect immunoflourecence antibody test	2008
Lin et al (78)	Toxoplasma	Taiwan		426	Hosptials	ELISA	2008
	IgG indigenous		40.60%				
	IgG Immigrant		18.20%				
	IgM indigenous		2.90%				
	IgM Immigrant		2.20%				
Ndiaye et al (79)	Toxoplasma	Senegal		109	No information	Immunoenzymatic technical in solid phase	2002
	IgG		22.00%				
	IgM		3.00%				
El Mansouri et al (80)	Toxoplasma	Morocco	50.60%	2456	No information	ELISA	2007

Castilho-Pelloso et al (81)	Toxoplasma IgM		1.07%	290	No information	ELISA	January 2001 to December 2003
Alvarado-Esquivel et al (71)	Toxoplasma IgG	Mexico	6.10%	343	No information	IMX toxo IgM and IMX toxo IgG 2.0 kits	2006
	Toxoplasma IgM		0.00%				
Razzak et al (82)	Toxoplasma IgM	Iraq	0.97%	310	No information	Enzyme-linked immunofluorescent assay	July 2002 till September 2003
Spalding et al (83)	Toxoplasma	Brazil	71.50%	2126	No information		2005
Ndir et al (84)	Toxoplasma pregnant	Senegal	37.10%	140 (70 pregnant and 70 abortion patient)	No information	Technical solid-phase enzyme immunoassay	November 2001 to April 2002
	Toxoplasma aborted		40.00%				
Buchy et al (85)	Toxoplasma IgG	Vietnam	11.20%	300	No information	0	2003
	Toxoplasma IgM		0.00%				
Elnahas et al (86)	Toxoplasma IgG	Sudan	34.10%	487	Antenatal clinic	Enzyme linked immunoassay	June through to December 2000
	Toxoplasma IgM		14.30%				
Akoijam et al (87)	Toxoplasma	India	41.75%	503	No information	ELISA	August 1996 and September 1997
Mahdi et al (88)	Toxoplasma	Iran	49.20%	254	No information	0	1999
Rai et al (89)	Toxoplasma	Nepal	55.20%	345	No information	Microlatex agglutination (MLA) and ELISA methods	1998
Chintana et al (90)	Toxoplasma IgG	0	13.20%	1200	No information	Sabin-feldman dye tes	1998
Ashrafunnessa Khatun et al (91)	Toxoplasma IgG	Bangladesh	38.50%	286	Antenatal clinic	ELISA	1988
Zhang et al (92)	Toxoplasma	China	7.28%	1250	No information	Indirect hemaglutination (IHA)	1997
Onadeko et	Toxoplasma	Nigeria	75.40%	0	No	Dye test on sera	1996

al (93)					information		
Zhang et al (94)	Toxoplasma IgM	China	3.38%	4126	Perinatal care	Capture-EIA	1997
Gonzalez-Morales et al (95)	Toxoplasma	Cuba	70.90%	0	No information	ELISA	1990 and 1991
Galvan Ramirez et al (96)	Toxoplasma IgG	Mexico	34.90%	350	High-risk pregnancies in hospital		1995
	Toxoplasma IgM		20.70%				
Lelong et al (97)	Toxoplasma	Madagas car	83.50%	599	No information	ELISA	1995
Sun et al (98)	Toxoplasma IgG	China	39.14%	1211	Hospitals	ELISA	1995
	Toxoplasma IgM		4.21%				
Martinez Sanchez et al (99)	Toxoplasma	Cuba	71.00%	362	No information	10 µL ultra micro-ELISA	October 1990 to April 1991
Bari et al (100)	Toxoplasma IgG	India	46.00%	320	No information	ELISA	1990
	Toxoplasma IgM		27.70%				
Natu et al (101)	Toxoplasma		19.44%	499	No information	Indirect haemagglutination test.	1989
Zhang et al (102)	Toxoplasma	China	4.06%	6822	No information	Indirect hemagglutination (IHA)	1989
Abdel-Hafez et al (103)	Toxoplasma aborted	Jordon	58.20%	55	No information	ELISA and indirect immunofluorescent (IIF) test (for aborting woman)	1986
	Toxoplasma pregnant		26.10%	46			
Shanmugam et al (104)	Toxoplasma	India	23.60%	225	Antenatal clinic	Passive haemagglutination test (PHA)	0
Reis et al (105)	Toxoplasma	Brazil	61.10%	10468	Hospitals	Fluorometric tests, with IgM capture	2006
Harma et al (106)	Toxoplasma IgG	Turkey	60.40%	1149	Prenatal clinic	ELISA	2004
	Toxoplasma	.	3.00%				

	IgM						
Hou et al (107)	Toxoplasma	China	5.50%	347	No information	Indirect haemagglutination (IHA)	March and August 1996
Doehring et al (108)	Toxoplasma	Tanzania	0.8% check result with Igor	849	No information	Sabin-feldman dye test (DT) and an immunosorbent agglutination assay	1995
Soto et al (109)	Toxoplasma	Venezuela	53.91%	7969	No information	Indirect haemagglutination	0
Franklin et al (110)	Toxoplasma	Israel	21.00%	213	Antenatal clinic	Immunofluorescent antibody test	1988-1989 (16 months)
Khurana et al (69)	Toxoplasma IgG	India	15.33%	300	No information	ELISA	January 2005 to 2006
	Toxoplasma IgM		3.00%				
Ouermi et al (111)	Toxoplasma IgG	Burkina Faso	27.20%	276 (HIV infected and uninfected pregnant women)	Healthcare facility	ELISA	January to June 2009
	Toxoplasma IgM		4.70%				
Hammouda et al (112)	Toxoplasma	Egypt	65.00%	100	No information	ELISA	1993
	CMV		51.00%				
