# Supplementary material for: Epidemiology and aetiology of maternal parasitic infections in low- and middle-income countries
Source: J Glob Health. 2011 Dec;1(2):189–200. (PMC3484768)
Supplement: Supplementary Table 4 [file jogh-01-189-s004.pdf]

Supplementary Table 4. Summary of data extracted from studies reporting prevalence of maternal infection with Chagas disease (n=7)

Author	Country	Prevalence	Numbers in study	Study setting	Tests used	Study dates
Araujo et al (4)	Brazil	0.30%	351	Patients	Antibodies in umbillical cord	2009
Arancibia et al (5)	Chile	2.10%	2063	Non-endemic area	Indirect immunoflourescence test (iift)	1991-1993
Arcavi et al (6)	Argentina	8.50%	729	Non-endemic area	Indirect hemagglutination and indirect immunofluorescence	Jan 1990- Feb 1991
Mendoza Ticona et al (7)	Peru	0.73%	3000	Endemic areas	Indirect immunofluorescence (iif), (elisa) testing and titration of immunoglobulin g (igg) antibodies by iif	December 2001 - July 2002
Sosa-Estani et al (8)	Argentina, Bolivia, Honduras, and Mexico	5.50%	2495	Endemic areas	ELISA	2008
Torrico et al (9)	Bolivia	26.30%	2124	Hospital	No information	2004
Arcavi et al (6)	Argentina	8.50%	729	No information	Indirect haemagglutination and indirect immunofluorescence tests.	January 1990 to February 1991
