# Supplementary material for: Epidemiology and aetiology of maternal parasitic infections in low- and middle-income countries
Source: J Glob Health. 2011 Dec;1(2):189–200. (PMC3484768)
Supplement: Supplementary Table 5 [file jogh-01-189-s005.pdf]

Supplementary Table 5 Summary of data extracted from studies reporting prevalence of maternal infection with trichinellosis (n=1)

Author	Pathogen	Country	Prevalence	Numbers in study	Study setting	Diagnostic test used	Year studied
Taybouavone. et al (118)	Trichinellosis	Lao Peoples' Democratic Republic	2.00%	200	No information	Western Blot	2006
