# Supplementary material for: Epidemiology and aetiology of maternal parasitic infections in low- and middle-income countries
Source: J Glob Health. 2011 Dec;1(2):189–200. (PMC3484768)
Supplement: Supplementary Table 6 [file jogh-01-189-s006.pdf]

Supplementary Table 6. Summary of data extracted from studies reporting prevalence of maternal infection with *Trichomonas vaginalis* (n=4)

Author	Pathogen	Country	Prevalence	Numbers in study	Study setting	Diagnostic test used	Year studied
Perazzi et al (113)	Trichomonas vaginalis	Argentina	4.00%	597	No information	culture on liquid medium	2005
Stringer et al (114)	Trichomonas vaginalis	Africa	18.00%	2428	No information	analysed data from HPTN 024	2010
Azargoon et al (115)	Trichomonas vaginalis	Iran	5.50%	1223	Hospital	vaginal pH, saline wet mount, and Amsel tests	2006
Ogbonna et al (116)	Trichomonas vaginalis	Nigeria	37.60%	250	Urban		2000
		.	24.80%	250	Rural		
Assefa et al (117)	Trichomonas vaginalis	Congo	18.60%	215 HIV +	No information	culture	2010
			10.20%	206 HIV -			
